# Assessment of Early Glaucomatous Optic Neuropathy in the Dog by Spectral Domain Optical Coherence Tomography (SD-OCT)

**DOI:** 10.3390/mi15060780

**Published:** 2024-06-13

**Authors:** Annie Oh, Christine D. Harman, Kristin L. Koehl, Jiayan Huang, Leandro B. C. Teixeira, Laurence M. Occelli, Eric S. Storey, Gui-Shuang Ying, András M. Komáromy

**Affiliations:** 1 Department of Small Animal Clinical Sciences, College of Veterinary Medicine, Michigan State University, East Lansing, MI 48824, USA; harmanc1@msu.edu (C.D.H.); koehlkri@msu.edu (K.L.K.); l_occelli@yahoo.fr (L.M.O.); 2 Department of Clinical Sciences, College of Veterinary Medicine, North Carolina State University, Raleigh, NC 27606, USA; 3 Department of Ophthalmology, Scheie Eye Institute, Perelman School of Medicine, University of Pennsylvania, Philadelphia, PA 19104, USA; huangjiayan@gmail.com (J.H.); gsying@pennmedicine.upenn.edu (G.-S.Y.); 4 Comparative Ocular Pathology Laboratory of Wisconsin, Department of Pathobiological Sciences, School of Veterinary Medicine, University of Wisconsin-Madison, Madison, WI 53706, USA; lteixeira@wisc.edu; 5 Department of Veterinary Clinical Sciences, School of Veterinary Medicine, Louisiana State University, Baton Rouge, LA 70803, USA; storeye@hotmail.com

**Keywords:** canine, glaucoma, SD-OCT, optic nerve head, retinal thickness

## Abstract

Background: Inherited primary open-angle glaucoma (POAG) in Beagle dogs is a well-established large animal model of glaucoma and is caused by a G661R missense mutation in the *ADAMTS10* gene. Using this model, the study describes early clinical disease markers for canine glaucoma. Methods: Spectral-domain optical coherence tomography (SD-OCT) was used to assess nine adult, *ADAMTS10*-mutant (median age 45.6 months, range 28.8–52.8 months; mean diurnal intraocular pressure (IOP): 29.9 +/− SEM 0.44 mmHg) and three related age-matched control Beagles (mean diurnal IOP: 18.0 +/− SEM 0.53 mmHg). Results: Of all the optic nerve head (ONH) parameters evaluated, the loss of myelin peak height in the horizontal plane was most significant (from 154 +/− SEM 38.4 μm to 9.3 +/− SEM 22.1 μm; *p* < 0.01). There was a strong significant negative correlation between myelin peak height and IOP (Spearman correlation: −0.78; *p* < 0.003). There were no significant differences in the thickness of any retinal layers evaluated. Conclusions: SD-OCT is a useful tool to detect early glaucomatous damage to the ONH in dogs before vision loss. Loss in myelin peak height without inner retinal thinning was identified as an early clinical disease marker. This suggests that initial degenerative changes are mostly due to the loss of myelin.

## 1. Introduction

Primary glaucoma is a leading cause of non-curable vision loss in dogs with an estimated prevalence of 0.89% [1,2]. The disease affects both eyes and is characterized by abnormal elevation of intraocular pressure (IOP), depression of the optic nerve head (ONH), and progressive death of retinal ganglion cells (RGCs) [3]. Primary closed-angle glaucoma (PCAG) is the most common form of the disease, where acute, severe increases in IOP are difficult to predict, and dogs are often diagnosed too late to rescue sight despite medical and surgical treatment options to lower IOP. It is suspected that intermittent IOP spikes occur before the diagnosis of the disease, leading to undetected early glaucomatous damage [4]. Therefore, early diagnosis of glaucoma is most important for effective therapy before more severe irreversible damage has occurred [5]. This includes a detailed quantitative assessment of the ONH, with the standard method being direct and indirect ophthalmoscopy. While advanced changes, such as ONH atrophy and cupping, are readily detectable, these methods are unreliable in detecting early and mild morphologic changes. Thus, more sensitive and powerful in vivo imaging techniques, including optical coherence tomography (OCT), have recently been developed for high-resolution imaging of the ocular fundus. 

OCT utilizes low-coherence interferometry to indirectly measure optical reflections to produce in vivo ocular images [6,7]. Spectral-domain optical coherence tomography (SD-OCT) is an advanced OCT system that is clinically available. It measures the echo time delay of light with an interferometer broad bandwidth light source and simultaneously captures all backscattered light frequencies using a spectrometer. Compared to the reference arm of time-domain (TD) OCT, simultaneous acquisition of light frequencies by SD-OCT permits rapid scanning (24,000–55,000 A-scans/s), higher axial resolution (3–6 μm), and fewer artifacts [8,9,10]. SD-OCT also permits further increases in sensitivity and deeper tissue penetration with the enhanced depth imaging (EDI) OCT function [11,12], which is particularly useful when analyzing posterior ocular structures.

OCT is the cutting-edge imaging technology for diagnosing, monitoring progression, and quantifying structural damage for glaucoma. In human patients, the retinal nerve fiber layer (RNFL) around the ONH is the most frequently used structural parameter. The addition of ONH parameters improves diagnostic accuracy for glaucoma detection [13,14]. Detailed studies about OCT for glaucoma diagnosis exist for dogs [4,15,16,17]. Interestingly, despite the high glaucoma prevalence in dogs, only one peer-reviewed publication utilized OCT to assess early glaucomatous retinal changes in several dog breeds with PCAG [4]. To date, there is no comprehensive OCT study about the early glaucomatous changes of the canine ONH. Because the canine ONH is unique, with myelinated RGC axons being present in the prelaminar ONH, parameters developed in other species, including human glaucoma patients, cannot be directly translated to the dog. 

The purpose of this study was to provide a detailed SD-OCT examination of the canine glaucomatous ONH with a special focus on developing the parameters of early markers for glaucomatous damage. To achieve our goal, we performed our study on a well-established form of the disease, primary open-angle glaucoma (POAG) in the Beagle dog. This disease has been thoroughly characterized in >90 publications published since 1972 [3,18]. Beagle POAG is an autosomal recessive trait caused by a G661R missense mutation in the *ADAMTS10* gene [19]. This condition is well suited for this study not only because the disease progresses more slowly and is more predictable than PCAG, with a gradual IOP increase, but also because the pathologic changes within the ONH have been very well defined at the structural and ultrastructural levels [20,21,22].

## 2. Materials and Methods

### 2.1. Animals

*ADAMTS10*-mutant dogs (*n* = 9; 6 males and 3 females; median age 45.6 months, range 28.8–52.8 months; mean diurnal IOP: 29.9 +/− SEM 0.44 mmHg) and control, related dogs of comparable age (*n* = 3; 2 males and 1 female; median age: 35.4 months; range 31.2–67.2 months; mean IOP: 18.0 +/− SEM 0.53 mmHg) were part of a canine POAG colony of Beagle-derived mongrel dogs carrying the G661R missense mutation (Table 1). Genotypes were confirmed by PCR, gel electrophoresis, and Sanger sequencing [19]. The dogs were housed under a 12-hour light:dark cycle, and a simple maze test confirmed the presence of vision. The study was conducted in compliance with the Association for Research in Vision and Ophthalmology statement for Use of Animals in Ophthalmic and Vision Research and approved by the Michigan State University (MSU) Institutional Animal Care and Use Committee (IACUC ID: PROTO202200005). 

### 2.2. Ophthalmic Examination

Regular ophthalmic examinations were performed pre- and post-imaging with SD-OCT. Anterior segments were examined for clarity and cells with diffuse and focal illumination using portable hand-held slit-lamp biomicroscopes (Kowa SL14; Kowa Company, Tokyo, Japan). Fundic examinations were performed with portable binocular indirect ophthalmoscopes (Keeler All Pupil II; Keeler Instruments, Broomall, PA, USA) and condensing lenses (Pan Retinal 2.2D; Volk Optical, Mentor, OH, USA). 

Normal iridocorneal angle morphology was confirmed by gonioscopy with a RetCam II (Clarity Medical Systems, Pleasanton, CA, USA) following ocular surface anesthesia (proparacaine HCl 0.5% ophthalmic solution; Alcon Laboratories, Inc., Fort Worth, TX, USA), and the use of a viscoelastic gel (OptixCare^®^ Plus Eye Lube; Aventix, Burlington, ON, Canada) and a gonioscopy lens. Axial globe length was manually measured from ocular images produced by A- and B-scan ultrasonography (Humphrey A/B scan system model 837, Humphrey Instruments, San Leandro, California). Diurnal IOPs (8 AM, 11 AM, 3 PM) were collected once monthly for 5 months by one examiner (CH) with a rebound tonometer (Icare^®^ TonoVet; Icare Finland Oy, Vantaa, Finland) [23].

### 2.3. Spectral-Domain Optical Coherence Tomography (SD-OCT) Image Acquisition

**Figure 1:**
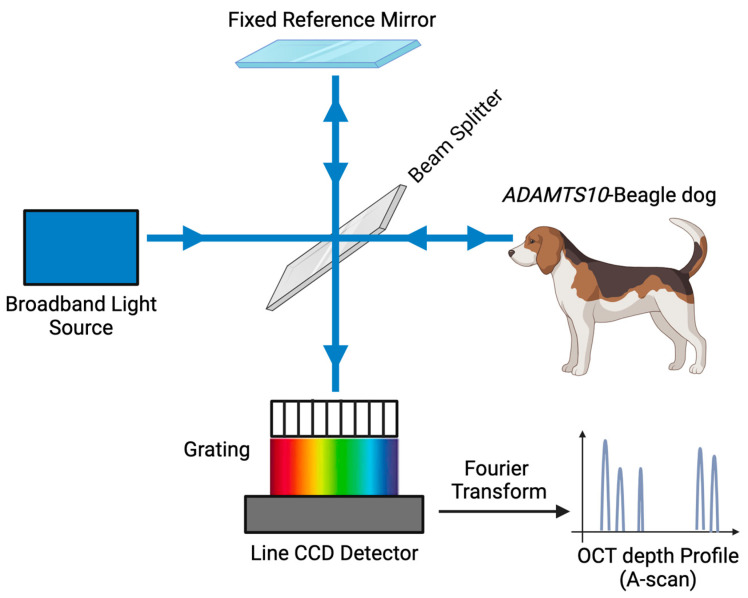
Schematic diagram of spectral-domain optical coherence tomography (SD-OCT). Line charge-coupled device (CCD) detector. (Referenced from Zheng S et al. [26]).

The pupils were dilated with topical tropicamide 1% ophthalmic solution USP (Akorn, Lake Forest, IL, USA). The dogs were placed under general anesthesia for optical image quality: premedicated with intramuscular acepromazine maleate (Butler Schein Animal Health, Dublin, OH, USA; 0.2 mg/kg), induced with intravenous propofol (PropoFloTM28, Abbott Laboratories, North Chicago, IL, USA; 4 mg/kg), intubated, and maintained on isoflurane/O_2_ gas mixture. They were placed in sternal recumbency. The position of the globes was maintained via 4-0 silk conjunctival stay sutures (Perma-hand, Ethicon, Somerville, NJ) placed at the superior and inferior limbus. SD-OCT with a 30° lens was used to image the canine fundus (Spectralis^®^, Heidelberg Engineering, Heidelberg, Germany) (Figure 1). Four different types of scans were used per eye: retinal nerve fiber layer (RNFL) 12° circle, line, radial, and volume (Table 2). A confocal scanning laser ophthalmoscope (cSLO) image was also acquired with each scan. Several parameter options could be chosen for each scan; this was dependent on the quality of the image produced by the SD-OCT. However, EDI-OCT was specifically utilized in the line and radial scans to enhance sensitivity and penetration of deeper tissues [24]. Each scan was centered on the ONH, and a real-time eye-tracking system of the SD-OCT stabilized the retinal position. At the end of the SD-OCT sessions, the stay sutures were removed, and the animals were allowed to awaken under close supervision. Drug-induced mydriasis was reversed in *ADAMTS10*-mutants with topical 0.005% latanoprost ophthalmic solution (Greenstone LLC., Peapack, NJ, USA) [25].

One masked investigator (AO) analyzed the cSLO image and SD-OCT scans through the HRA/Spectralis^®^ Viewing Module (version 5.4.6.0) and ImageJ (NIH ImageJ; NIH, Bethesda, MD, USA). The units were in µm or pixels^2^. 

#### 2.3.1. Optic Nerve Head (ONH) Measurements

**Figure 2:**
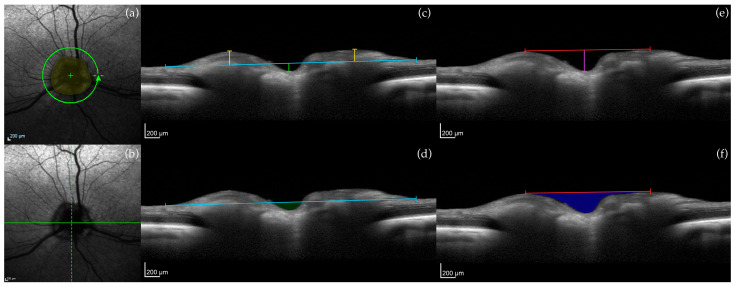
Example of optic nerve head (ONH) measurements in a control dog. (**a**) cSLO image showing the location of the circular 12° OCT RNFL scan: ONH area (yellow shade); (**b**) cSLO image of a line scan: 0° (solid green line) and 90° (dashed green line) line scans; (**c**) Vitreoretinal reference plane (blue line): myelin peak height (yellow line) and ONH cup depth (green line). (**d**) Vitreoretinal reference plane (blue line): cross-sectional area of the ONH cup (green shade). (**e**) Neuroretinal rim reference plane (red line): myelin peak distance (length of red line) and ONH cup depth (purple line). (**f**) Neuroretinal rim reference plane (red line): cross-sectional area of the ONH cup (blue shade). Calibration bars = 200 μm.

On the cSLO image of an RNFL scan, the ONH area was determined (Figure 2a; yellow shade). Because the canine ONH protrudes more noticeably into the vitreous compared to humans [27], two distinct methods of measurement were used to measure canine ONH structures (Table 3). 

In the first approach, vitreoretinal and neuroretinal reference planes were used. On a line scan, 0° (horizontal) and 90° (vertical) line scans were selected (Figure 2b; 0° = solid green line, 90° = dashed green line), and the vitreoretinal reference plane was created by drawing a horizontal line along the vitreoretinal surface (Figure 2c; blue line) with the center of the line matched with the deepest point of the ONH cup. Following, ONH cup depth (Figure 2c; green line), myelin peak height (Figure 2c; yellow line), and cross-sectional area of the ONH cup (Figure 2d; green shade) were measured. Similarly, on 0° and 90° line scans, the neuroretinal reference plane was established by drawing a horizontal line between the two highest points of the ONH (Figure 2e; red line). From here, ONH cup depth (Figure 2e; purple line), myelin peak distance (Figure 2e; red line), and cross-sectional area of the ONH cup (Figure 2f; blue shade) were measured.

**Figure 3:**
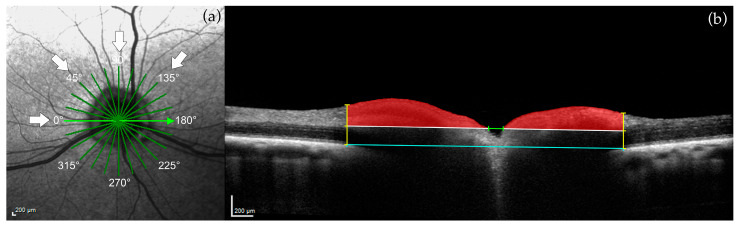
Example of optic nerve head (ONH) measurements in a control dog. (**a**) cSLO image showing the location of the 12 standard radial scans: four of these line scans were selected for measurements (white arrows; 0°, 45°, 90°, 135°). (**b**) Connected ends of the retinal pigment epithelium/choriocapillaris layer (blue line). One-hundred fifty micrometer reference plane (white line). Optic cup diameter (green line). Perpendicular lines from the ends of the retinal pigment epithelium/choriocapillaris layer to the inner limiting membrane (yellow lines). Cross-sectional neuroretinal rim area (red shade). Calibration bars = 200 μm.

The second approach was adapted from Schuman et al. [28]; ONH parameters determined from the 150 µm reference plane on SD-OCT detected early glaucomatous changes in humans [29]. Four line scans were selected from the standard 12 radial scans imaged (Figure 3a; white arrows). The ends of the retinal pigment epithelium/choriocapillaris layer were identified and connected with a straight line (Figure 3b; blue line). Anteriorly by 150 µm, a second line was drawn to create the 150 µm reference plane (Figure 3b; white line). From this plane, optic cup diameter (Figure 3b; green line) and cross-sectional neuroretinal rim area (Figure 3b; red shade) were measured. 

#### 2.3.2. Retinal Layer Measurements

**Figure 4:**
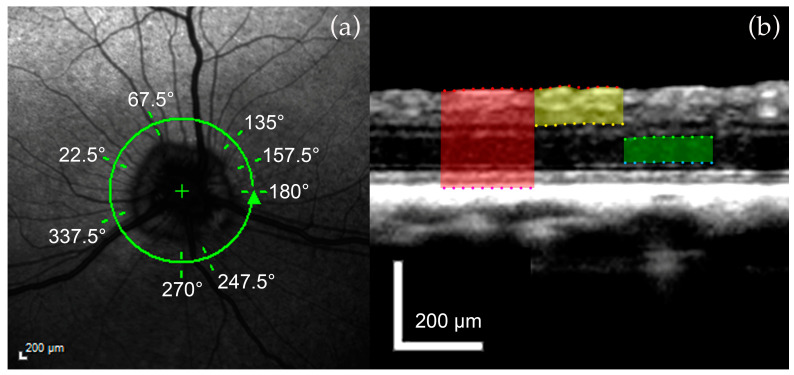
Example of retinal layer measurements in a control dog. (**a**) cSLO image of the 12° circular RNFL OCT scan: two distinct points per quadrant void of retinal blood vessels were identified. (**b**) FRT (red shade) extends from the ILM (red dots) to the RPE (purple dots). IRT (yellow shade) extends from the ILM (red dots) to the posterior surface of the IPL (yellow dots). ONL (green shade) is measured from its anterior (green dots) to the posterior surface (blue dots). Calibration bars = 200 μm.

On an RNFL scan, peripapillary full retinal thickness (FRT), inner retinal thickness (IRT), and outer nuclear layer (ONL) were measured (Table 3). In detail, the RNFL scan was divided into four quadrants, and two distinct points per quadrant void of retinal blood vessels were used (Figure 4a). Areas containing retinal blood vessels were avoided, as vessels contribute to artificial thickening of the retinal layers [30]. FRT was automatically calculated, while IRT and ONL were manually determined by correcting the delineation of the individual layers (Figure 4b).

### 2.4. Statistical Analysis

A total of 61 measurements were obtained from cSLO and SD-OCT images of the ONH and retina from each eye. ANOVA was used to compare the dimensions of the ocular structures between POAG-affected and control dogs. Spearman correlation coefficient was applied to determine the association of the SD-OCT values with age, IOP, and axial globe length since these three parameters are positively associated with POAG progression [3]. For all tests, *p* ≤ 0.05 was significant. 

### 2.5. Tissue Collection and Histology

Age-matched dogs (*n* = 4) were selected from the wider canine POAG colony to compare histology with SD-OCT images. The dogs were euthanized with an overdose of sodium pentobarbital for reasons unrelated to this study. Following enucleation, the globes were fixed in Karnovsky’s paraformaldehyde-glutaraldehyde solution for routine processing, paraffin embedding, and histologic evaluation of H&E stained sections.

## 3. Results

### 3.1. Optic Nerve Head

**Figure 5:**
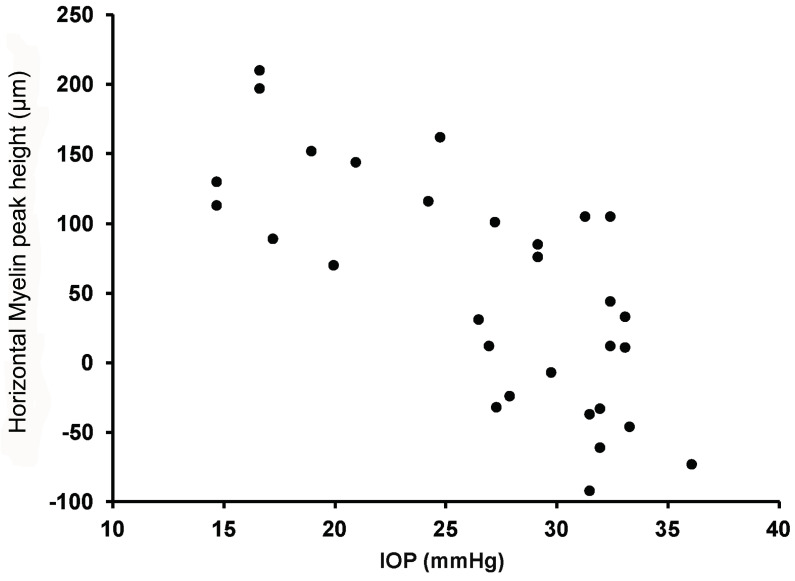
Scatterplot showing a significant negative correlation between intraocular pressure (IOP) and myelin peak height.

SD-OCT data sets were obtained from both eyes of 12 Beagle dogs. There was a significant difference in myelin peak height between the *ADAMTS10* mutants (*n* = 9) and controls (*n* = 3). The loss of myelin in the mutant dogs was present in both 0° (horizontal) and 90° (vertical) line scans, but it was most significant in the 0° line scans (from 154 +/− 38.4 μm to 9.3 +/− 22.1 μm; *p* < 0.01; Table 4). Moreover, there was a strong, significant negative correlation between myelin peak height and IOP (Figure 5) that was also pronounced in the 0° line scans (Spearman correlation coefficient: −0.78; *p* < 0.003; Table 4). 

There was no significant correlation between myelin peak height and age in both 0° (Spearman correlation coefficient: −0.04; *p* = 0.90) and 90° (Spearman correlation coefficient: −0.14; *p* = 0.66) line scans. There was no significant correlation between myelin peak height and axial globe length in both 0° (Spearman correlation coefficient: −0.53; *p* = 0.08) and 90° (Spearman correlation coefficient: −0.41; *p* = 0.18) line scans. Furthermore, there was no significant difference in axial globe length between *ADAMTS10*-mutant (21.7 +/− SEM 0.25 μm) and control dogs (20.8 +/− SEM 0.23 μm).

**Figure 6:**
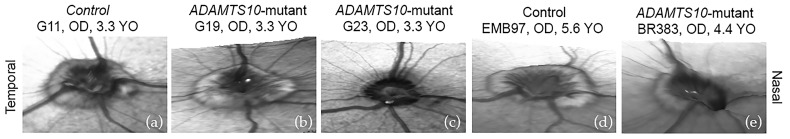
Volume scans of three-dimensional SD-OCT images of the ONH. Two normal (**a**,**d**) and three affected ONHs (**b**,**c**,**e**) at various ages and stages of disease are shown. Right eye (OD); years old (YO).

On volume scans, three-dimensional views between *ADAMTS10* mutants and controls reveal that ONH cupping was rather severe in a few animals (Figure 6c,e), but the cup dimensions measured in this study did not significantly differ between groups. When comparing three mutant and two control ONHs at various ages, there was variability in the disease stage and glaucomatous damage, particularly in young *ADAMTS10* mutants (Figure 6b,c).

**Figure 7:**
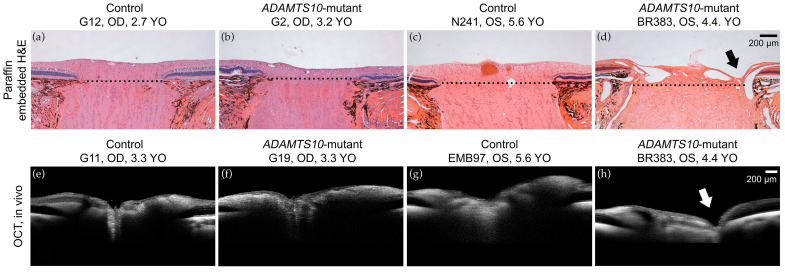
Histologic and SD-OCT comparison of the ONH. Normal (**a**,**c**,**e**,**g**) and age-matched mutant Beagle dogs (**b**,**d**,**f**,**h**). The 3.2-year-old and 3.3-year-old mutant dogs exhibit mild flattening of myelin on histology (**b**) and SD-OCT (**f**), respectively, in contrast to the 4.5-year-old mutant dog, which has marked myelin loss and cupping (arrows) of the ONH on both histology (**d**) and SD-OCT (**h**). Calibration bars = 200 μm. Right eye (OD); Left eye (OS); years old (YO).

SD-OCT cross-sectional images of a 3.3-year-old mutant dog also appear rather unremarkable, with mild flattening of myelin (Figure 7f), while histologic cross sections show thinning of the pre-laminar ONH (Figure 7b). At an older age (4.4-year-old), SD-OCT images demonstrate cupping of the ONH (Figure 7h; white arrow), and myelin loss. Similar changes are observed on histologic evaluation (Figure 7d; black arrow) with the addition of posterior dislocation of the lamina cribrosa (dashed line). In vivo SD-OCT images of control dogs at various ages also illustrate the characteristic “volcano-like” bulge of the ONH into the vitreous; this is not apparent on histologic cross sections of either mutants or controls.

### 3.2. Retinal Layers

There were no significant differences in the thickness of the peripapillary retinal layers evaluated between the *ADAMTS10* mutant and control groups (Table 5), as mean FRTs (+/− SEM) were 239 μm (+/− 6.0 μm) and 243 μm (+/− 3.2 μm), respectively.

**Figure 8:**
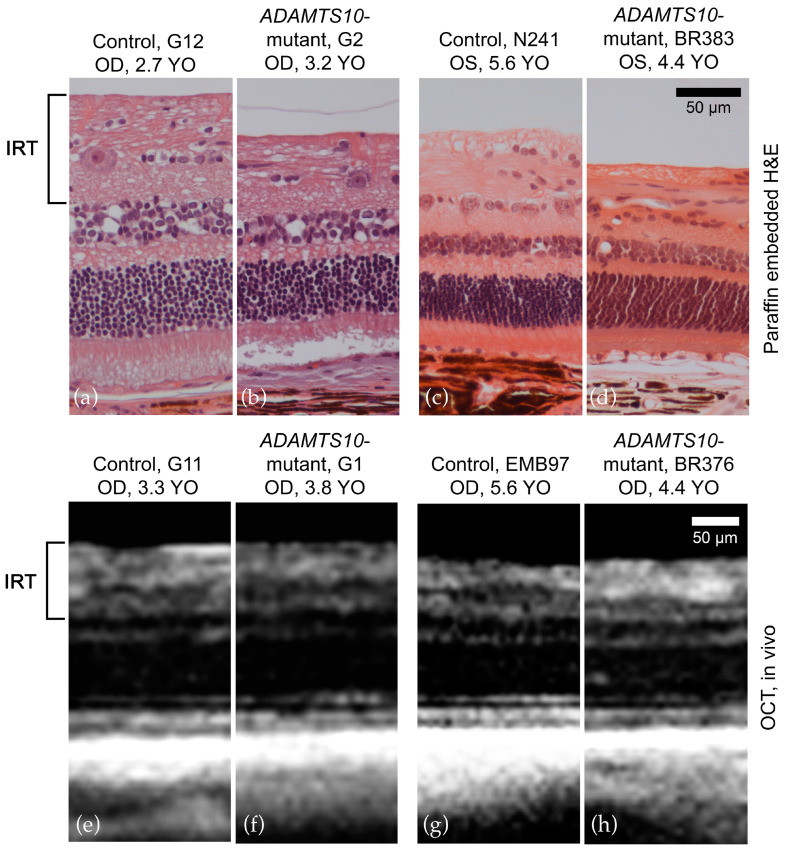
Histologic (superior retina) and SD-OCT (peripapillary) comparison of the retina. Normal (**a**,**c**,**e**,**g**) and age-matched mutant Beagle dogs (**b**,**d**,**f**,**h**). On histologic sections, there was thinning of the inner retina in the affected tissues (**b**,**d**) compared to age-matched controls (**a**,**c**) in both the early and later stages of the disease. This was not observed on SD-OCT when comparing the affected tissues (**f**,**h**) with age-matched controls (**e**,**f**) at the ages assessed. Calibration bars = 50 μm. Right eye (OD); Left eye (OS); years old (YO).

Histologic cross sections of the superior retina depict thinning of the inner retina in the affected tissues compared to the age-matched controls in both early and later periods of the disease (Figure 8). This was not apparent in the SD-OCT images at the disease stages evaluated.

## 4. Discussion

The purpose of this study was to describe early glaucomatous optic neuropathy in POAG-affected dogs with an in vivo imaging modality. This is a well-established and clinically relevant animal model of human POAG [3]. Our data support that SD-OCT can detect early ONH changes in *ADAMTS10*-mutant Beagles, the key result being significant myelin loss in the horizontal plane because of elevated IOP. Severe ONH cupping was observed in a few older animals, but neither the average dimensions of the optic cup nor the thickness of the retina (FRT, IRT, and ONL) varied significantly between the mutant and control groups. 

Significant loss of myelin is a sensitive quantitative measurement of ONH degeneration. The ONH is an intrapapillary equivalent to the RNFL [31] and composed of RGC axons [21]. The viscoelastic myelin of the RGC axons forms the raised and irregularly shaped ONH in the dog [27], and primary demyelination results in progressive loss of ONH architecture [21,22,32]. In our study, we identified this region as myelin peak height, but it can also be known as neuroretinal rim height. The horizontal loss of neuroretinal rim height observed in our colony (Table 4) was previously described in POAG-affected Beagles [33]. A consistent pattern of rim loss in glaucomatous dogs, however, has not been observed.

The loss of myelin peak height (from 154 ± 38.4 μm to 9.3 ± 22.1 μm; *p* < 0.01) is a consequence of elevated IOP (Figure 5; Spearman correlation: −0.78; *p* < 0.003). The canine ONH is prominent after birth [31], and its morphology is comparable between 5.6-month-old control and age-matched pre-glaucomatous dogs. As IOP progressively increases from the normal range of 10–20 mmHg [32,33] in 8- to 16-month-old POAG-affected Beagles, ultrastructural modifications of the optic nerve axons [34,35], and slight compression and distortion of the lamina cribrosa [21] lead to early architectural ONH lesions. These insults cannot be identified clinically with direct or indirect ophthalmoscopic examination [21,33]. However, with SD-OCT, significant myelin peak loss was observed both quantitatively (Table 4) and qualitatively (Figure 6). 

ONH cupping was rather severe in a few older animals, but the average cup dimensions did not significantly differ between the control and mutant Beagles. The raised canine ONH may have contributed to the lack of difference in optic cup measurements between the two groups. In several radial scans of normal and mildly affected *ADAMTS10*-mutant Beagles, the optic cup was located above the 150 μm reference plane, and the optic cup diameter could not be identified. ONH cupping was only present in severely affected *ADAMTS10*-mutant dogs, and thus a further study comparing control and moderately to severely affected *ADAMTS10*-mutant Beagles is required. In particular, individuals should be selected according to their average IOP, rather than to factors such as age or axial globe length. Our data revealed that neither age nor axial globe length significantly contributed to changes in ONH architecture in *ADAMTS10*-mutant Beagles. 

SD-OCT revealed no significant difference in peripapillary thickness of FRT and IRT between the control and *ADAMTS10*-mutant dogs. ONL thickness was also consistent between normal and glaucomatous eyes, as neither cone loss nor increased photoreceptor thickness was seen in the parafoveal region, as seen in previous studies [36,37]. Peripapillary FRT and IRT thinning occurs as a consequence of ocular hypertension reducing axoplasmic flow of neurotrophins and perfusion to optic nerve axons, ultimately leading to RGC death [21,34,35]. Thinning of the RNFL with SD-OCT was identified as an early glaucomatous change in several dog breeds with PCAG [4] and corresponds to areas of structure and visual field loss in human patients [38,39]. The discrepancy in early clinical disease markers between POAG in Beagles and PCAG in other dog breeds may be linked to how IOP increases in each disease: slow and progressive in POAG, and acute and severe in PCAG. But also, Beagles with *ADAMTS10* mutation have a mechanically weak posterior sclera associated with reduced collagen density and a lower proportion of insoluble collagen [40], thus potentially resulting in changes to the ONH before the retina.

One limitation of this study is the small sample size, rendering our results preliminary. Even though the *ADAMTS10*-mutant Beagles were visual and had comparable IOP measurements, there was variability in the disease stage and glaucomatous damage in the *ADAMTS10*-mutant Beagles. A larger sample size would have decreased the influence of variability on the results. This will be achieved by collecting more data in the future. Additionally, the optic cup diameter was not measured in several normal and mildly affected *ADAMTS10*-mutant Beagles because the optic cup was located above the 150 μm reference plane. Furthermore, the repeatability of the scanning technique and the reliability of observer measurements were not explored. Granted reliability of observer measurements, specifically intra-rater reliability, could not have been assessed, as POAG progresses over time.

## 5. Conclusions

SD-OCT successfully detects the ultrastructural change in *ADAMTS10*-mutant Beagles. Myelin peak height is the most sensitive quantitative measurement of early glaucomatous optic neuropathy and should be observed in future longitudinal SD-OCT studies. Average optic cup measurements did not significantly change, but SD-OCT scans of a cohort of moderately to severely affected dogs may prove otherwise. Peripapillary retinal thickness is not a core parameter for the assessment of POAG-affected dogs.

## Figures and Tables

**Table 1:** Profiles of Study Subjects.

Canine ID	Sex	Age (Years)	POAG Status	IOP (mmHg)		Axial Length (mm)
M685	M	2.6	−	OD/OS	14.7/16.6	20.9/20.5
G11	M	3.3	−	OD/OS	20.9/18.9	20.9/20.9
EMB97	F	5.6	−	OD/OS	19.9/17.2	20.1/20.1
BLSU076	M	2.4	+	OD/OS	32.4/29.1	21.1/22.1
G19	M	3.3	+	OD/OS	27.2/26.5	21.9/21.2
G23	F	3.3	+	OD/OS	31.9/33.3	20.4/21.3
G1	M	3.8	+	OD/OS	31.3/32.4	21.1/22.1
G3	M	3.8	+	OD/OS	29.7/26.9	21.8/22.8
G4	M	3.8	+	OD/OS	36.1/31.9	21.7/21.3
G18	M	3.8	+	OD/OS	27.9/27.3	20.5/21.0
BR383	F	4.4	+	OD/OS	31.5/33.1	23.4/24.0
BR376	F	4.4	+	OD/OS	24.2/24.7	21.0/21.2

Male (M), female (F); primary open-angle glaucoma (POAG) status: affected (+), control (−); mean diurnal intraocular pressure (IOP): right eye (OD), left eye (OS).

**Table 2:** SD-OCT Scan Parameters.

	RNFL Scan	Line Scan	Radial Scan	Volume Scan
**Scan Type**	Circle	Single	Volume	Volume
**Resolution Mode**	HR or HS	HR or HS	HR or HS	HR or HS
**Scan Angle**	30°	30°	15° or 30°	20° or 30°
**Scan Width**		30°	15° or 30°	
**Pattern Size**				20° × 20° or 30° × 25°
**Circle Diameter**	12°			
**ART**	up to 100 images averaged	up to 100 images averaged	up to 16 images averaged	up to 16 images averaged
**EDI Mode**	Off	On	On	Off
**Number of B-Scans**			12	49 or 61
**Angle between B-Scans**			15°	
**Distance between B-scans**				122 µm

Retinal nerve fiber layer (RNFL) scan; high resolution (HR), high-speed mode (HS); Automatic Real-Time (ART); Enhanced Depth Imaging (EDI)-mode.

**Table 3:** ONH and Retinal Layer Measurements.

ONH Measurements	Retinal Layer Measurements
ONH area	FRT
*Vitreoretinal reference plane* ONH cup depth, myelin peak height, cross-sectional area of the ONH cup	IRT
*Neuroretinal rim reference plane*	ONL
ONH cup depth, myelin peak distance, cross-sectional area of the ONH cup	
*150 μm reference plane*	
Optic cup diameter, neuroretinal rim area	

Optic nerve head (ONH); Full retinal thickness (FRT), inner retinal thickness (IRT), outer nuclear layer (ONL).

**Table 4:** Statistically Significant Results of ONH Parameters.

SD-OCT Parameter	Line Scans	Control (*n* = 3)	Mutant (*n* = 9)	*p*-Value	Correlation with IOP	*p*-Value
Myelin peak height	0°	154 +/− 38.4 μm	9.3 +/− 22.1 μm	0.010	−0.78	0.003
	90°	119 +/− 30.9 μm	24.0 +/− 17.7 μm	0.026	−0.57	0.05

Spectral-domain optical coherence tomography (SD-OCT); 0° (horizontal) and 90° (vertical) line scans; Correlation with mean intraocular pressure (IOP).

**Table 5:** Statistical Results of Retinal Parameters.

SD-OCT Parameter	Location	Control (*n* = 3)	Mutant (*n* = 9)	*p*-Value
Average FRT	Peripapillary	239 +/− 6.0 μm	243 +/− 3.2 μm	0.510
Average IRT	Peripapillary	89.4 +/− 4.4 μm	90.8 +/− 2.7 μm	0.790
Average ONL	Peripapillary	56.4 +/− 2.9 μm	52.9 +/− 3.9 μm	0.640

Spectral-domain optical coherence tomography (SD-OCT); full retinal thickness (FRT); inner retinal thickness (IRT); outer nuclear layer (ONL).

## Data Availability

The raw data supporting the conclusions of this article will be made available by the authors on request.
